# Complete mitochondrial genome of *Odoiporus longicollis* (Coleptera: Curculionidae) and phylogenetic analysis

**DOI:** 10.1080/23802359.2025.2582875

**Published:** 2025-10-31

**Authors:** Haonan Sun, Shengtao Xu, Kesuo Yin, Xundong Li, Arslan Jamil, Zhixiang Guo, Lina Liu

**Affiliations:** The Ministry of Agriculture and Rural Affairs Key Laboratory for Prevention and Control of Biological Invasions, Agricultural Environment and Resources Institute, Yunnan Academy of Agricultural Sciences, Kunming, China

**Keywords:** *Odoiporus longicollis*, mitogenome, phylogenetic analysis

## Abstract

*Odoiporus longicollis* (Marshall, 1930), a major banana pest in the family Curculionidae, primarily infests banana pseudostems. The mitogenomic analysis of *O. longicollis* and its phylogenetic relationships enhances the available mitogenomic data of Curculionidae and provides valuable resources for the molecular identification, population monitoring, and management of this species. In this study, the complete mitogenome of *O. longicollis* (16,294 bp) was sequenced for the first time. The circular genome comprises 13 protein-coding genes (PCGs), 22 tRNAs, and two rRNAs, with GC contents of 24.81%. Phylogenetic analysis based on 16 Curculionidae mitogenomes offers novel insights into the evolutionary relationships of *O. longicollis*.

## 1. Introduction

*Odoiporus longicollis,* a diminutive beetle belonging to the Curculionidae family within Coleoptera (CABI 2022), is widely distributed across southwestern China. It constitutes one of the most significant biotic constraints in commercial banana cultivation, posing significant threats to banana yield (Alagersamy et al. 2016). The larvae of *O. longicollis* possess a reddish-brown head and a pale yellowish-white, plump and apodous body. Upon metamorphosis, the adult beetles display a slender body with a predominantly reddish-brown coloration. *O. longicollis* primarily infests banana pseudostems, leading to yield losses ranging from 10% to 90% in banana cultivation (Alagesan et al. 2018). Significant economic losses attributed to *O. longicollis* have been reported, with infestation levels varying considerably across banana cultivars. For instance, the maximum infestation rate observed in Nendran bananas is 31.69% (Preetha et al. 2023). As dynamic organelles, mitochondria regulate essential processes including metabolic flux modulation, transitions between developmental stages, and cytochrome c-mediated apoptosis within insect systems (Dong et al. 2021). To date, no published reports on the mitogenome assembly of *O. longicollis* are available in scientific databases. In the current study, the complete mitogenome of *O. longicollis* was sequenced using the Illumina NovaSeq X platform, and its phylogenetic relationships were analyzed.

## 2. Materials and methods

**Figure 1.**
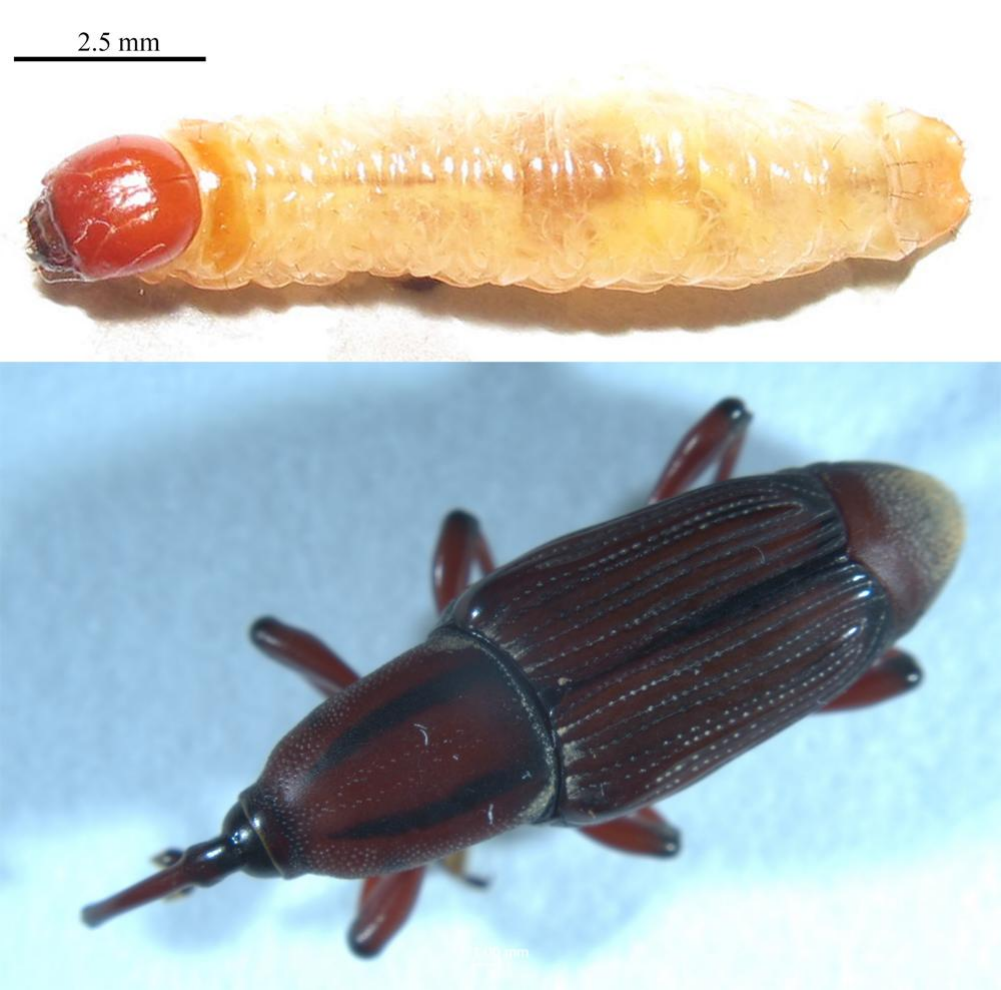
Morphological appearance of *O. longicollis* larva and adult (voucher number: Y001). Photographed by Lina Liu.

Adult specimens of *O. longicollis* were collected from Xishuangbanna State, Yunnan Province, China (22°0′38.7′′N, 10°47′45.6′′E), deposited at Agricultural Environment and Resources Institute, Yunnan Academy of Agricultural Sciences under ultra-low temperature conditions (http://www.iaer.cn/), and assigned the voucher number Y001 (contact Lina Liu, handyliu@126.com). Based on its collection from the banana pseudostem and morphological characteristics, the specimen was identified by the corresponding author (Singh 2018). The larvae and adults of *O. longicollis* are presented in Figure 1.

Total DNA from *O. longicollis* (mixed popularion) was extracted using a modified CTAB protocol (Allen et al. 2006). The purified DNA was then sent to Tsingke (Beijing, China) for whole genome library construction using the NEBNext^®^ Ultra^™^ DNA Library Prep Kit for Illumina^®^. Sequencing was performed using the Illumina NovaSeq X platform, a whole-genome shotgun strategy was used to perform paired-end sequencing of the whole-genome library, with read lengths of 150 bp. Raw sequencing reads were preprocessed using SOAPnuke version 1.3.0 to trim adapters and remove low-quality data (Chen et al. 2018). Genome assembly was performed using SPAdes version 3.13.0, and the assembled sequences were aligned against closely related reference mitogenome, the final assembly was gap-filled by Gapcloser Version: 1.12 (Bankevich et al. 2012; Luo et al. 2012). *O. longicollis* gap-free mitogenome assembly spans 16,294 bp (NCBI accession: NC_062876). Genome architecture was annotated by MITOS2 (http://mitos2.bioinf.uni-leipzig.de/index.py), and a visual output of the circular mitogenome was displayed (Bernt et al. 2013).

Phylogenetic analysis was conducted using thirteen mitogenomes from the Curculionidae family and three outgroup mitochondrial genomes from the Cerambycidae family, all retrieved from the NCBI GenBank database. Thirteen additional mitogenomes belonging to various subfamilies of Curculionidae were obtained from GenBank through BLAST searches using the assembled *O. longicollis* mitogenome. Thirteen universal PCGs and the *COX1* gene from the 16 species were extracted, aligned, and concatenated using PhyloSuite (version 1.2.3) (Zhang et al. 2020). PartitionFinder2 (version 2.1.1) was employed to determine the optimal evolutionary model (Lanfear et al. 2017). The final results was analyzed using MrBayes (version: 3.2.7a) and IQ-TREE (version: 2.2.0) for Bayesian Inference (BI) and Maximum Likelihood (ML) methods, respectively (Ronquist et al. 2012; Minh et al. 2020). BI analyses were conducted under default MCMC settings (Generations = 2,000,000, Sampling Freq = 1,000, Nrun = 2, Nchains = 4, Contype = Allcompat, Conformat = Simple, Burnin Fraction = 0.25) and terminate-d when the effective sample size above 100. In ML analyses, the bootstrap typ-e was ultrafast and with 5,000 replicates. Finally, the phylogenetic tree was vi-sualized by iTOL (https://itol.embl.de/) (Letunic and Bork 2021).

## 3. Results

**Figure 2.**
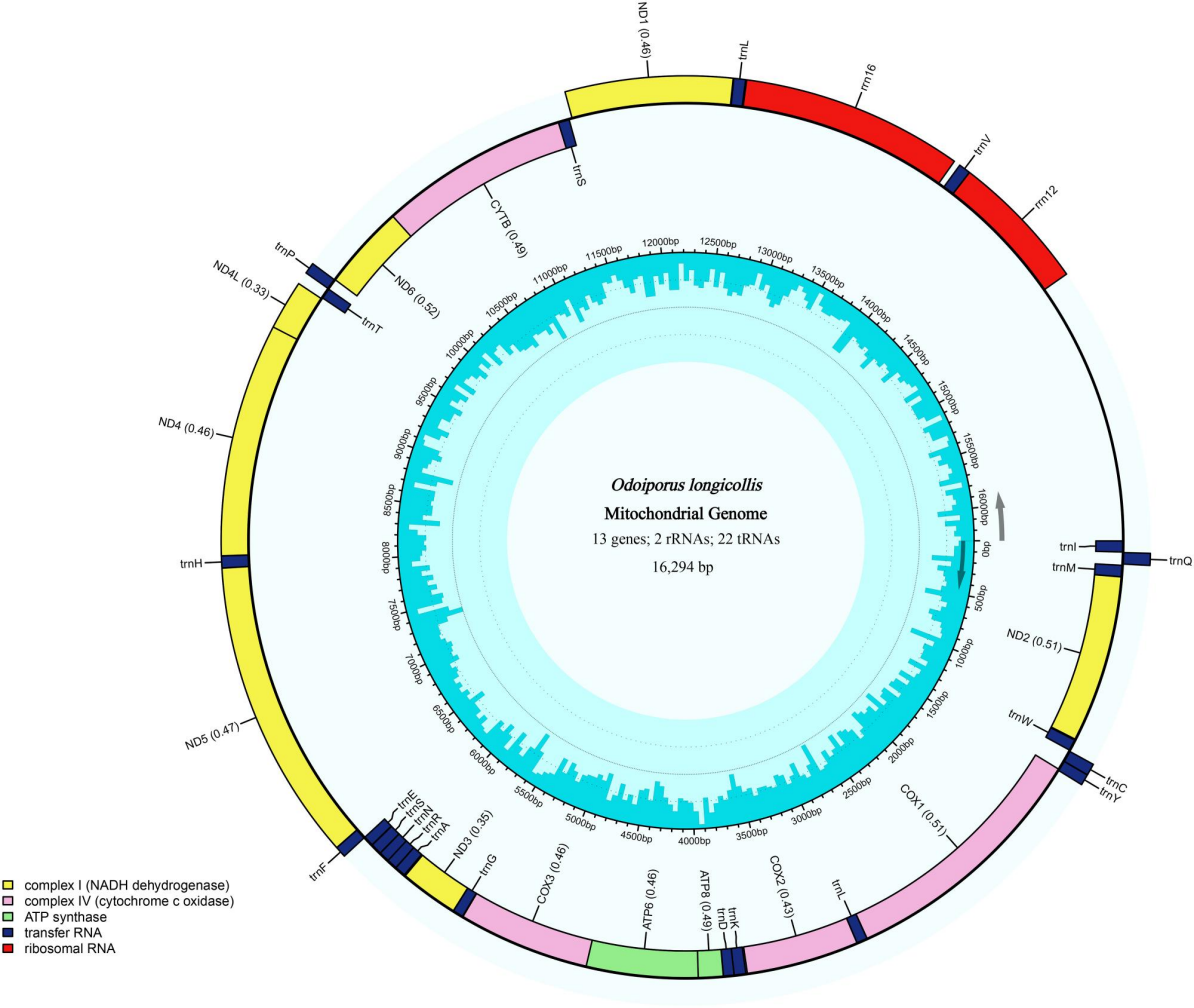
The complete mitogenome map of *O. longicollis*. The innermost ring of the figure shows GC content. The outer ring displays the mitochondrial DNA structure of *O. longicollis.*

The complete mitogenome of *O. longicollis* is circular and spans 16,294 bp in length. It comprises 13 core PCGs (ND2, COX1, COX2, ATP8, ATP6, COX3, ND3, ND5, ND4, ND4L, ND6, CYTB, ND1), 22 tRNA genes and two rRNA genes. No introns, cis-splicing and trans-splicing genes were detected among the annotated genes. The contents of A, C, G, and T are 39.25%, 16.79%, 8.02%, and 35.90%, respectively, with a GC content of 24.81%. Among the 13 PCGs, six PCGs (ATP6, COX3, ND5, ND4, ND4L, CYTB) utilize ATG as the initiation codon, the remaining PCGs exhibit non-canonical initiation codon, including ATC (COX2, ATP8, ND3, ND6), ATT (ND2, COX1), ATA (ND1). Two types of termination codons were observed: twelve PCGs terminate with TAA, whereas ND1 uniquely uses TAG as its stop codon (Figure 2, Table S1).

**Figure 3.**
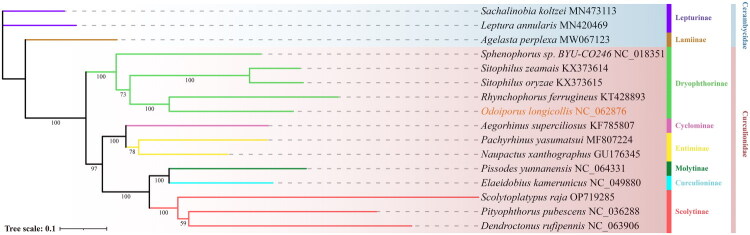
Maximum likelihood (ML) phylogenetic tree of *O. longicollis* and other species of the order coleoptera based on sequences of 13 PCGs. The GenBank accession number from NCBI are provided after the species names. The following sequences were used: MN473113 (Nie et al. 2021), MN420469 (Pu et al. 2022), MW067123 (Li et al. 2021), NC_018351 (Song et al. 2010), KX373614 (Ojo et al. 2016), KX373615 (Ojo et al. 2016), KT428893 (Roberts et al. 2024), KF785807 (Cabrera-Brandt and Gaitán-Espitia 2015), MF807224 (Zhang et al. 2017), GU176345 (Song et al. 2010), NC_064331 (unpublished), NC_049880 (Apriyanto and Tambunan 2020), OP719285 (Yu et al. 2023), NC_036288 (Meng et al. 2023), NC_063906 (Meng et al. 2023). Orange sample represents the target species of this study. Numbers on nodes refer to ML bootstrap values. The scale bar refers to 0.1 nucleotide substitutions per character.

Maximum likelihood (ML) phylogenetic analysis of the complete mitogenome indicated that the thirteen Curculionidae species were resolved into three distinct clades, and the resulting topology was congruent with their established taxonomic classification. The ML phylogenetic tree based on the *COX1* gene was entirely consistent with the complete mitogenome tree at the major clade level, with only minor differences observed in the branching of individual species (Figure S1). Moreover, compared with inter-subfamily species, those within the same subfamily exhibited lower nucleotide divergence in the *COX1* gene, indicating a lower degree of genetic differentiation within subfamilies. *O. longicollis* was recovered as the closest relative of *Rhynchophorus ferrugineus* (Figure 3). Notably, the species composition of each clade and branch in the ML phylogenetic tree was identical to that observed in the Bayesian inference (BI) tree (Figure S2, Figure S3).

## 4. Discussion

In this study, the complete mitochondrial genome of *O. longicollis* was characterized, and phylogenetic analysis further enriched the mitogenomic dataset of Curculionidae. Recent investigations have reported mitogenomes from various Curculionidae taxa, including *Scolytoplatypodini* and *Curculiochinensis*, demonstrating conserved gene arrangements and comparable evolutionary rates across PCGs (Hu et al. 2021; Yu et al. 2023). Coleoptera mitogenomes exhibit the conserved architecture of closed circular double-stranded DNA molecules, typically ranging in size from 14,257 bp to 21,628 bp (Bae et al. 2004; Kim et al. 2015), with size variation primarily attributed to differences in the presence and number of introns (Li et al. 2021). The mitochondrial genome of *O. longicollis* is 16,294 bp in length, falling within the documented size range for Coleopteran mitogenomes, and notably contains no introns. Seven PCGs in this genome employ non-canonical initiation codons (ATC/ATT/ATA), representing a deviation from the standard mitochondrial genetic code typically observed in Coleoptera (Nie 2014; Sheffield et al. 2008).

Furthermore, this study elucidated the phylogenetic relationships within Curculionidae. Among the three reciprocally monophyletic clades inferred from thirteen Curculionidae species, *O. longicollis* formed a sister-group relationship with *R. ferrugineus* (NCBI accession: KT428893.1). These two species clustered within the same branch of the phylogenetic tree, whose members are classified morphologically within the subfamily Dryophthorinae. Accordingly, the mitochondrial-based phylogenetic findings are congruent with traditional morphological classification (Chamorro et al. 2021). Aside from its slightly larger body size, *R. ferrugineus* shares a largely identical morphology with *O. longicollis* (Singh 2018; Chamorro 2019; Hoddle et al. 2024). Reports of *R. ferrugineus* infesting banana plantations, exhibiting damage to banana pseudostems indistinguishable from that caused by *O. longicollis*, suggest that there may be niche overlap or sharing between these two species (Gargi et al. 2025; Kalita et al. 2023). To date, no studies have reported a genetic relationship between *O. longicollis* and *R. ferrugineus* from a bioinformatic perspective, this study is the first to propose such an association.

## Supplementary Material

Supplementary figures and tables.docx

## Data Availability

The mitogenome sequence data that support the findings of this study are openly available in GenBank of NCBI (https://www.ncbi.nlm.nih.gov/) under the accession NC_062876. The associated BioProject, SRA, and BioSample numbers are PRJNA1259690, SRR33454076, and SAMN48369847 respectively.
