# Supplementary material for: Complete mitochondrial genome of Odoiporus longicollis (Coleptera: Curculionidae) and phylogenetic analysis
Source: Mitochondrial DNA B Resour. 2025 Oct 31;10(12):1083–7. doi: 10.1080/23802359.2025.2582875 (PMC12581775; doi:10.1080/23802359.2025.2582875)
Supplement: Supplementary figures and tables.docx [file TMDN_A_2582875_SM2887.docx]

**Table S1.** Gene organization of the mitochondrial genome in *Odoiporus longicollis*

| Gene | Position | | Length | | Codon | |
| --- | --- | --- | --- | --- | --- | --- |
|  | From | To | nt | aa | Start | Stop |
| *trnI-GAT* | 1 | 67 | 67 | - | - | - |
| *trnQ-TTG* | 70 | 138 | 69 | - | - | - |
| *trnM-CAT* | 146 | 214 | 69 | - | - | - |
| *nad2* | 215 | 1222 | 1005 | 335 | ATT | TAA |
| *trnW-TCA* | 1228 | 1295 | 68 | - | - | - |
| *trnC-GCA* | 1301 | 1368 | 68 | - | - | - |
| *trnY-GTA* | 1369 | 1432 | 64 | - | - | - |
| *cox1* | 1425 | 2969 | 1545 | 515 | ATT | TAA |
| *trnL2-TAA* | 2965 | 3029 | 65 | - | - | - |
| *cox2* | 3030 | 3707 | 693 | 231 | ATC | TAA |
| *trnK-CTT* | 3716 | 3786 | 71 | - | - | - |
| *trnD-GTC* | 3786 | 3850 | 65 | - | - | - |
| *atp8* | 3851 | 4006 | 156 | 52 | ATC | TAA |
| *atp6* | 4000 | 4671 | 672 | 224 | ATG | TAA |
| *cox3* | 4671 | 5462 | 792 | 264 | ATG | TAA |
| *trnG-TCC* | 5465 | 5528 | 64 | - | - | - |
| *nad3* | 5529 | 5882 | 342 | 114 | ATC | TAA |
| *trnA-TGC* | 5890 | 5955 | 66 | - | - | - |
| *trnR-TCG* | 5956 | 6019 | 64 | - | - | - |
| *trnN-GTT* | 6019 | 6082 | 64 | - | - | - |
| *trnS1-TCT* | 6083 | 6150 | 68 | - | - | - |
| *trnE-TTC* | 6152 | 6214 | 63 | - | - | - |
| *trnF-GAA* | 6214 | 6277 | 64 | - | - | - |
| *nad5* | 6278 | 7994 | 1692 | 564 | ATG | TAA |
| *trnH-GTG* | 7995 | 8062 | 68 | - | - | - |
| *nad4* | 8063 | 9392 | 1323 | - | ATG | TAA |
| *nad4l* | 9386 | 9667 | 282 | - | ATG | TAA |
| *trnT-TGT* | 9674 | 9738 | 65 | - | - | - |
| *trnP-TGG* | 9739 | 9803 | 65 | - | - | - |
| *nad6* | 9806 | 10315 | 492 | 164 | ATC | TAA |
| *cob* | 10315 | 11454 | 1140 | 380 | ATG | TAA |
| *trnS2-TGA* | 11453 | 11519 | 67 | - | - | - |
| *nad1* | 11538 | 12485 | 924 | 308 | ATA | TAG |
| *trnL1-TAG* | 12489 | 12555 | 67 | - | - | - |
| *rrnL* | 12561 | 13818 | 1258 | - | - | - |
| *trnV-TAC* | 13855 | 13922 | 68 | - | - | - |
| *rrnS* | 13923 | 14710 | 788 | - | - | - |


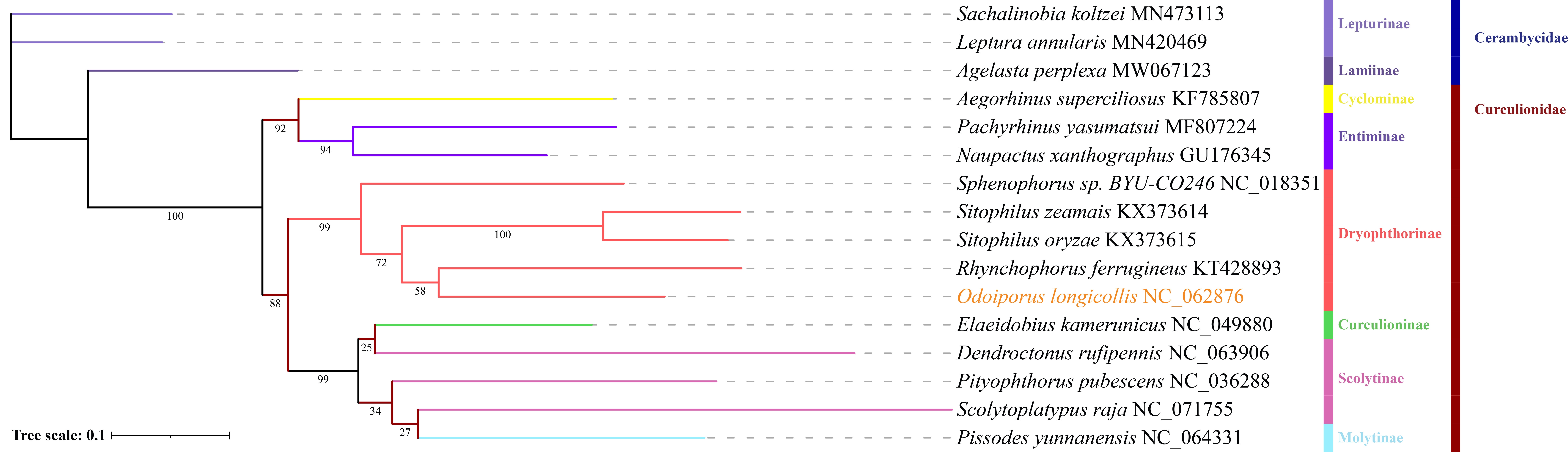


**Figure S1.** Maximum likelihood (ML) phylogenetic tree of *O. longicollis* and other species of the order Coleoptera based on sequences of *COX1* gene. The GenBank accession number from NCBI are provided after the species names. Orange sample represents the target species of this study. Numbers on nodes refer to ML bootstrap values. The scale bar refers to 0.1 nucleotide substitutions per character.


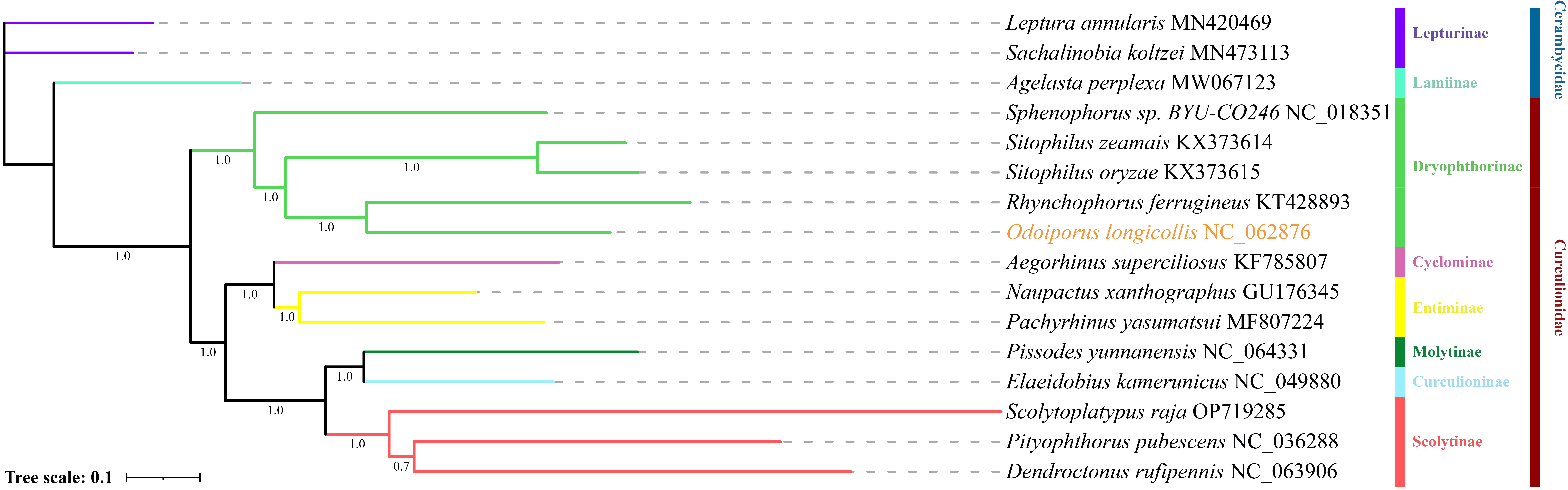


**Figure S2.** Bayesian inference (BI) phylogenetic tree of *O. longicollis* and other species of the order Coleoptera based on sequences of 13 PCGs. The GenBank accession number from NCBI are provided after the species names. Orange sample represents the target species of this study. Numbers on nodes refer to BI bootstrap values. The scale bar refers to 0.1 nucleotide substitutions per character.


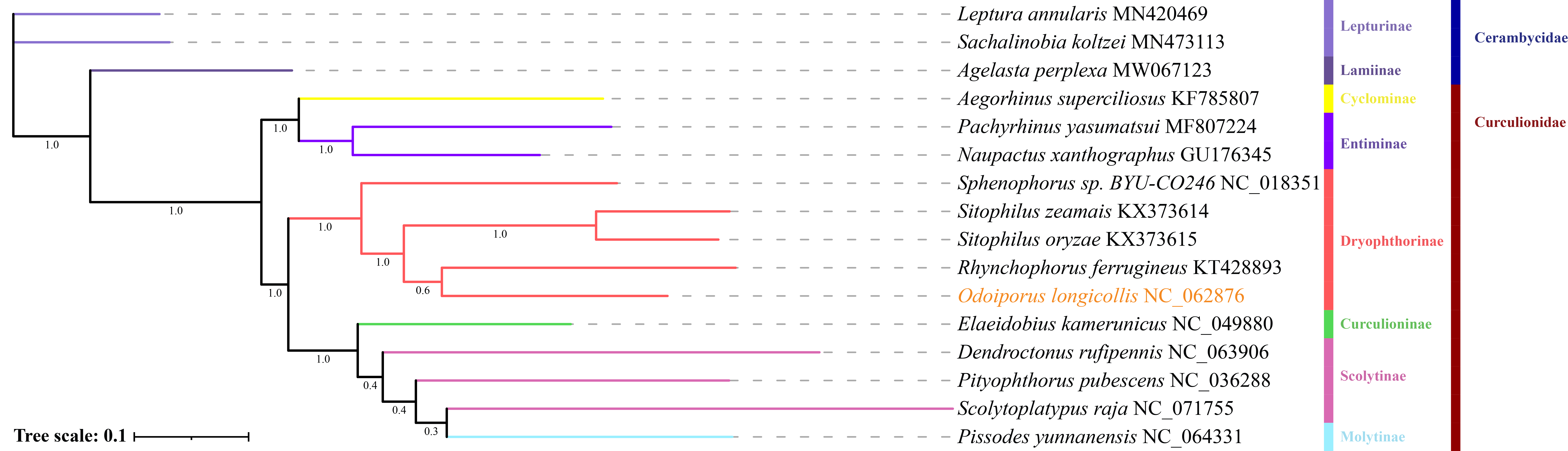


**Figure S3.** Bayesian inference (BI) phylogenetic tree of *O. longicollis* and other species of the order Coleoptera based on sequences of *COX1* gene. The GenBank accession number from NCBI are provided after the species names. Orange sample represents the target species of this study. Numbers on nodes refer to BI bootstrap values. The scale bar refers to 0.1 nucleotide substitutions per character.
